# Enhancing our conceptual understanding of state and trait self-efficacy by correlational analysis of four self-efficacy scales in people with spinal cord injury

**DOI:** 10.1186/s40359-020-00474-6

**Published:** 2020-10-19

**Authors:** Tijn van Diemen, Ashley Craig, Ilse J. W. van Nes, SELF-SCI Group, Charlotte van Laake, Jos Bloemen, Janneke Stolwijk-Swuste, Eline Scholten, Willemijn Faber, Joke Boerrigter, Martine Beurskens, Dorien Spijkerman, Karin Postma, Esther Groenewegen, Govert Snoek, Iris Martens, Ilse van Nes, Tijn van Diemen, Ellen Roels, Joke Sprik, Janneke M. Stolwijk-Swuste, Jan H. B. Geertzen, James Middleton, Marcel W. M. Post

**Affiliations:** 1 Department of spinal cord injury Rehabilitation, Sint Maartenskliniek, P.O. box 9011, 6500 GM Nijmegen, The Netherlands; 2 Center of Excellence for Rehabilitation Medicine, UMC Utrecht Brain Center, University Medical Center Utrecht, and De Hoogstraat Rehabilitation, Utrecht, the Netherlands; 3 University of Groningen, University Medical Center Groningen, Department of Rehabilitation Medicine, Hanzeplein 1, 9713 GZ Groningen, The Netherlands; 4 John Walsh Center for Rehabilitation Research, Northern Clinical School, Faculty of Medicine and Health, The University of Sydney, Kolling Institute, St Leonards, NSW Australia; 5 Department of spinal cord injury, De Hoogstraat Rehabilitation, Utrecht, the Netherlands; 6 Spinal Outreach Service, Royal Rehab, Sydney, Australia

**Keywords:** Spinal cord injuries, Self-efficacy, Rehabilitation, Validation study, Outcome measures

## Abstract

**Background:**

Self-efficacy is an important determinant of adjustment following spinal cord injury. Self-efficacy is defined as the belief that one can successfully execute behavior required to produce the desired outcomes. In its original conceptualization, self-efficacy refers to the confidence that people have in their ability to accomplish specific tasks and behaviors within a specific context. Over the years these situation specific aspects have been unconfined and multiple constructs of self-efficacy have been proposed. The most common is a division in trait and state self-efficacy. Another used division that is utilized is between general, domain-specific and task-specific self-efficacy. The scientific support for these constructs is to date still unclear. The objective of this study was to enhance the understanding of the self-efficacy construct by comparing four self-efficacy scales designed to measure three aspects of self-efficacy (general versus domain-specific versus task-specific) in people with spinal cord injury.

**Methods:**

Dutch and Australian adults with spinal cord injury (*N* = 140) completed four frequently used self-efficacy scales; the Moorong Self-efficacy Scale, General Self-efficacy Scale, University of Washington Self-efficacy Scale and a Self-care Self-efficacy Scale approximately 6 months after their inpatient rehabilitation. Pearson correlations examined inter-relationships between the scales.

**Results:**

Hypothesized strong correlations between scales measuring similar aspects of self-efficacy were found (correlations 0.50–0.65). However, the hypothesized weak to moderate correlations between scales measuring diverging aspects of self-efficacy were only partly found (correlations 0.31–0.74), with 7 out of 12 correlations being strong instead of moderate.

**Conclusions:**

The expected distinctions between the three aspects of self-efficacy was not demonstrated. All four scales measure a common latent construct, most likely general self-efficacy aspects. Further research is necessary to find ways to improve the measurement of domain-specific and task-specific aspects of SE, so that they are sensitive enough to capture change over time, and thus enhance clinical outcomes of people with SCI as they adjust to their disability.

## Background

Spinal cord injury (SCI) affects both physical and psychological functioning and challenges all areas of a person’s life. Physical aspects include limitations in strength, function and mobility, loss of sensation, spasm, pain, and changes in bladder, bowel and sexual functioning [1]. These effects are associated with increased dependence on caregivers, reduced social and work participation and diminished quality of life [2–6]. Psychological consequences can include elevated depressive mood, anxiety and fatigue, which also may have a negative influence on quality of life [5, 7–10].

In recent years, there has been increased clinical and research interest in the contribution of self-efficacy (SE) in people with SCI [5, 11–13]. Self-efficacy is defined as the belief that one can successfully execute behavior required to produce the desired outcomes [14]. The SCI Adjustment Model or SCIAM [15], incorporating aspects of social learning, stress and coping, and health belief theories, views SE not just as an indicator or predictor of the potential to adjust, but also as a key component in the person’s appraisal/reappraisal process, labelled the “engine room” of the capacity to change, and thereby as a crucial element for adjustment and coping. In research, SE has been shown to be a key determinant of adjustment after SCI [13, 16–19], as well as in other chronic health conditions [20, 21]. Self-efficacy is therefore a valuable clinical predictor of adjustment of people with SCI, with strong correlations with depressed mood, anxiety, participation and quality of life [5, 16, 17, 22]. Arguably, self-efficacy could be a promising target for interventions during the SCI rehabilitation process [23].

In its original conceptualization, SE refers to the confidence that people have in their ability to accomplish specific tasks and behaviors within a specific context. This can be seen as ‘state-like’ SE, believed to be open to change over time as circumstances in someone’s life change, and believed to be modifiable by training or treatment [24]. However, over time, the concept of SE developed into a more general construct, namely the non-specific confidence people may have in managing their life in both routine and novel situations, and believed to be less open to change [24]. In a systematic review on the assessment of SE, Sheer [25] described three types of SE scales: trait-like or general SE (e.g., general stable belief in one’s ability to accomplish goals); domain-specific SE (e.g., a belief concerning managing aspects of chronic illness); and task-specific SE (e.g., a belief that is context bound involving a specific behavior). The last two are a further differentiation of the state aspect described above into domain and task-specific aspects of SE. State and trait constructs have been widely used in many areas of psychology and the distinction between them has been of considerable importance in psychological theory and research [26].

In SCI-related research, as well as in other areas of research, efforts have been made to measure different aspects of SE, which most likely overlap to some extent [17, 27, 28]. General SE is often measured in SCI research using one of the General Self-efficacy Scales (GSES) [19, 29–31]. A scale that has been used to measure domain-specific aspects of SE in people with SCI and multiple sclerosis is the University of Washington Self-efficacy Scale (UW-SES) [32, 33]. This scale was designed to measure the person’s ability to manage the consequences of their chronic health condition in their daily activities and social life. More recently, a new task-specific scale has been developed to measure self-care SE, the confidence that people with SCI have in their ability to perform appropriate self-care behaviors, called the Self-care Self-efficacy Scale (SCSES) [28].

The most commonly used SE scale in SCI research is the Moorong Self-efficacy Scale (MSES) [34]. This scale was originally designed to measure state aspects of SE. The factor structure of the MSES has been investigated several times over the years with two-factor and three-factor solutions found [34–37]. In the latest study, three subscales were identified namely: general SE (MSES-General), social functioning SE (MSES-Social), and personal functioning SE (MSES-Personal) [35], explaining 61% of the variance. The MSES-General obviously is marked as a scale measuring general-SE. The other two scales were found to be SCI-specific, this is state aspects of SE. The three factors showed strong correlations with corresponding aspects of other scales, supporting the validity. The found factor structure shows parallel with the distinctions between general, domain-specific and task-specific SE, respectively.

However, the validity and inter-relationships between these four scales, all designed to measure distinct aspects of SE, have not yet been empirically tested in SCI research. Consequently, the rationale of this study was to gather additional evidence about the construct validity of SE, using the abovementioned scales. It is also unclear how the results of studies using these different scales are interrelated. This position needs to be resolved if SE scales are to be employed usefully in clinical practice following SCI. Therefore, the aim of this current study was to clarify the construct of SE by examining the inter-relationships between the four abovementioned SE scales. The hypotheses for this study were: (i) SE scales that are intended to measure the same aspects of SE, that is general aspects of SE (measured with MSES-General and with GSES) or domain specific aspects of SE (measured with MSES-Social and with UW-SES) or task specific aspects of SE (measured with MSES-Personal and with SCSES) will show strong inter-correlations (> 0.5); (ii) SE scales that measure different aspects of SE, that is, general SE (MSES-General and GSES) versus domain-specific SE (MSES-Social and UW-SES) versus task-specific SE (MSES-Personal and SCSES) will show small to moderate inter-correlations (between 0.3 and 0.5), as the constructs are arguably different but not fully independent.

## Methods

### Participants

For this study, people with SCI were recruited in the Netherlands and Australia. The recruitment of the Dutch cohort has been described in detail elsewhere [28]. In short, 285 people with SCI, admitted to one of the eight specialized SCI rehabilitation centers in the Netherlands between January 2016 and January 2018, participated in this longitudinal cohort study. To be eligible to enter the study, participants were admitted for their initial rehabilitation after the occurrence of SCI, were 18 years of age and older, had no severe cognitive impairment due to comorbid brain injury according to medical records, no severe mental health disorders (such as schizophrenia) and possessed sufficient knowledge of the Dutch language to complete the self-reported questionnaires reliably. For the Australian cohort, participants in a SCI Outreach program in Sydney, New South Wales, who were between 3 and 9 months post-discharge after completing their subacute inpatient SCI rehabilitation in June 2018, were asked to take part in this study. The same inclusion criteria were applied, other than participants in this case needed to possess sufficient knowledge of the English language to complete the self-reported questionnaires.

### Procedure

In the Netherlands, the SE scales were part of the regimen of questionnaires in a longitudinal study. Data from the 6-months post-discharge assessment was used because the four SE scales were only administered together at this test occasion. Data collection took place between June 2017 and December 2018. Injury characteristics were recorded by an experienced rehabilitation physician at admission or retrieved from medical files. The Medical Ethics Committee of the University Medical Centre Utrecht declared that the study did not need formal ethical approval under the Dutch law regulating medical research in human beings (reference number: 15–449/C). After that, in accordance with local requirements, the Medical Ethics Committees of all participating Dutch rehabilitation centers approved the conduct of the study in their center.

In the Australian arm of the study, participants attending the Spinal Outreach Service in Sydney, were invited by postal mail to participate in a survey including the four SE questionnaires. In the case of no response (to participate or opt out) being received, a postal reminder was sent out 4 weeks after the first letter and the participants were telephoned after 8 weeks. Injury characteristics were retrieved from medical files. The Human Research Ethics Committee of the Northern Sydney Local Health District approved the project (reference number: LNR/17/HAWKE/269). The study, in both countries, was carried out in accordance with the code of conduct formulated in the Helsinki code. All participants provided informed consent before entering the study.

### Measures

Socio-demographic variables collected included age, sex, presence of a partner, educational level and pre-injury employment. Injury characteristics included: time since injury; traumatic or non-traumatic etiology; paraplegia or tetraplegia; and motor complete (i.e., AIS grades A and B) or motor incomplete (AIS grades C and D) lesion, according to the International Standards for Neurological Classification of Spinal Cord Injury (ISNCSCI) [38].

The MSES is a 16-item scale assessing an individual’s perception of control over their behavior for achieving their desired outcomes in relation to their SCI. Responses are provided on a 7-point Likert scale, ranging from 1 (very uncertain) to 7 (very certain). The MSES total scores range from 16 to 112, with higher scores indicating stronger SE. The MSES was translated into Dutch for this study using a translation – back translation procedure [39]. The MSES has been found to be a reliable and valid measure for people with SCI [34]. In the most recent validation study, [35] three factors were found, namely: General self-efficacy (e.g., Question 10: I can deal with unexpected problems that come up in life), Social functioning self-efficacy (e.g., Question 7: I can enjoy spending time with my friends) and Personal functioning self-efficacy (e.g., Question 1: I can maintain my personal hygiene with or without help) [35]. The internal consistency of the total MSES, in that recent validity study, was excellent (Cronbach α = .91), while the internal consistency of the MSES-General, MSES-Social and MSES-Personal factors were acceptable to good (Cronbach α = .81, .77 and .80, respectively) [35]. These figures were similar in the current study (total MSES .91; MSES-General .75; MSES Social .74, MSES Personal .75).

The Sherer General Self-efficacy Scale (GSES) was used to measure general SE [40]. The Dutch version of this scale has 12 items (of the original 17 items) [30]. Each item can be answered using a 5-point Likert scale ranging from 1 (strongly disagree) to 5 (strongly agree). The scores on this scale range from 12 to 60, where higher scores indicate stronger general self-efficacy. The same 12 questions were administered in the Australian cohort. An example of a question is: “When I make plans, I am certain I can make them work”. The Dutch GSES has been examined for its reliability and validity [41]. A re-examination of the original GSES version of Sherer, as well as the Dutch 12 item version, found three factors representing aspects of GSE, Initiative, Effort and Persistence [27, 30]. One of these studies, [30] demonstrated an improved fit with a higher order (one-factor) model, which therefore was chosen for this study. The internal consistency of the total score was good (Cronbach α = .85) in the current study.

Domain-specific SE was assessed using the short version of the University of Washington Self-efficacy Scale (UW-SES) [32]. This 6-item version has a 5-point scale ranging from 1 (not at all confident) to 5 (totally confident). The scale scores range between 6 and 30, with higher scores indicating greater disability-management SE. An example question is: “How confident are you that you can keep the physical discomfort of your spinal cord injury from interfering with the things you want to do?”. The UW-SES has been validated for people with SCI and multiple sclerosis and found to be reliable [32, 33, 42]. Prior research investigating the internal validity of the UW-SES 6 item version with confirmatory factor analysis and Rasch analysis has confirmed the one-factor structure [42, 43]. The internal consistency of the UW-SES 6 item version was excellent (Cronbach α = .90) in the current study.

For assessment of task-specific SE an adapted version of the Managing Disease in General subscale of the Self-efficacy for Managing Chronic Disease Scale [44] was used, referred to as the Self-Care Self-efficacy Scale (SCSES). Some items of this scale were changed to assess task-specific SE specific for SCI. This scale consists of 5 items with a 0–10 numeric rating scale indicating to what extent participants believe in their capacity to self-manage their health. The scale score ranges between 0 and 50, with higher scores indicating greater confidence in a person’s ability for self-care. The internal consistency of the original scale is good (Cronbach α = .87) [44]. The first two questions were not altered, asking about confidence in “doing all things necessary to manage the condition on a regular basis” and being able to “judge when to see a doctor”. The other three questions were adapted to read: 3 “How confident are you that you can keep up your physical condition and weight?”; 4 “How confident are you that you can prevent problems like pressure sores or urinary tract infections?”; and 5 “How confident are you that you can do everything necessary, in order to get the right aids and medication?”. The SCSES questionnaire can be found as Supplementary file 1. Cronbach α was good (0.81) in the current study.

### Statistical analyses

A power analysis with alpha = .05, small effect size of .25, power of 80%, 2 -tailed correlation, estimated a required sample size of 123. Missing items were replaced with the mean score of the (sub)scale. For the MSES Total score, less than 8% of the participants had one or more missing items that needed to be replaced. For the MSES-Social less than 5% and for all other scales less than 2.5%. Three participants had too many missing items to calculate one or more valid (sub) scores, these (sub) scores were marked as missing. Differences in socio-demographic and SCI characteristics between the two arms of the study (the Dutch and Australian participants) were tested using independent sample t-tests. Associations between the four scales, (e.g., MSES with three subscales, GSES, UW-SES and SCSES), the socio-demographic and SCI characteristics were calculated and expressed in Pearson correlations; with correlations up to 0.3 considered weak, between 0.3 and 0.5 as moderate and > 0.5 as strong [45]. All analyses were conducted using SPSS for Windows (version 25) (IBM corp, Armonk, NY).

## Results

In the Dutch arm of the study, a total of 119 participants completed all four SE scales. In the Australian arm, 21 of the 37 potential participants agreed to participate and completed all questionnaires. The characteristics of the participants are shown in Table 1.

**Table 1:** Characteristics of the study sample

Characteristic	Netherlands	N	Australia	N
Age in years Mean (SD)	55.1 (15.1)	119	60.4 (16.3)	21
Range	19–84		29–81	
Median (IQR)	56 (47–66)		64 (51.5–73.5)	
Sex, male N (%)	79 (66.4)	119	12 (57.1)	21
Having a partner N (%)	85 (73.3)	116	14 (66.7)	21
Education, bachelor degree or higher N (%)	41 (36.3)	113	7 (33.3)	21
Paid employment prior to SCI N (%)	83 (69.7)	119	9 (50.0)	18
Time since injury in weeks Mean (SD)	44.4 (12.5)*	119	55.9 (21.1) *	21
Range	30–95		27–103	
Median (IQR)	41 (36–49)		47 (40.5–70)	
Level of injury, paraplegia N (%)	57 (47.9)	119	10 (47.6)	21
SCI motor complete N (%)	29 (24.6)*	118	8 (38.1)*	21
SCI traumatic cause N (%)	65 (54.6)	119	13 (61.9)	21

* *P* < 0.05 according the independent samples t-test

Abbreviations: *SD* Standard deviation, *IQR* Interquartile range

Only time since injury and motor completeness of the SCI were significantly different between the cohorts (*p* < .05). A sensitivity test with only the Dutch cohort did not reveal a different pattern. Given the two cohorts were similar, they were combined for further analysis. The mean scores of the different SE scales are shown in Table 2.

**Table 2:** Mean scores of the four Self-efficacy scales, including the sub-scales of the Moorong Self-efficacy Scale, Cronbach’s alpha and the percentage of participants with the maximum scores on the scale

Measure (score range)	N	M (SD)	Median	IQR	Maximum score (%)
MSES Total (16–112)	138	86.5 (17.1)	90	75.8–100.0	1.4
MSES-General (4–28)	139	21.8 (4.4)	23	19.0–25.0	5.0
MSES-Social (5–35)	138	28.5 (5.5)	29	24.4–33.2	16.7
MSES-Personal (4–28)	138	21.7 (5.7)	23	19.0–26.0	15.9
GSES (12–60)	140	47.2 (8.3)	49	42.0–54.0	2.9
UW-SES (6–30)	139	17.5 (5.5)	18	13.0–22.0	1.4
SCSES (0–50)	139	38.6 (7.1)	40	35.0–44.0	7.2

Abbreviations: *M* Mean, *SD* Standard deviation, *IQR* Interquartile range, *MSES total* Moorong Self-efficacy Scale total score, *MSES-General* Moorong Self-efficacy Scale General factor, *MSES-Social* Moorong Self-efficacy Scale Social functioning self-efficacy factor, *MSES-Personal* Moorong Self-efficacy Scale personal functioning self-efficacy factor, *GSES* General Self-efficacy Scale, *UW-SES* University of Washington Self-efficacy Scale, *SCSES* Self-care Self-efficacy Scale

Correlations between the SE scales and determinants are shown in Table 3.

**Table 3:**
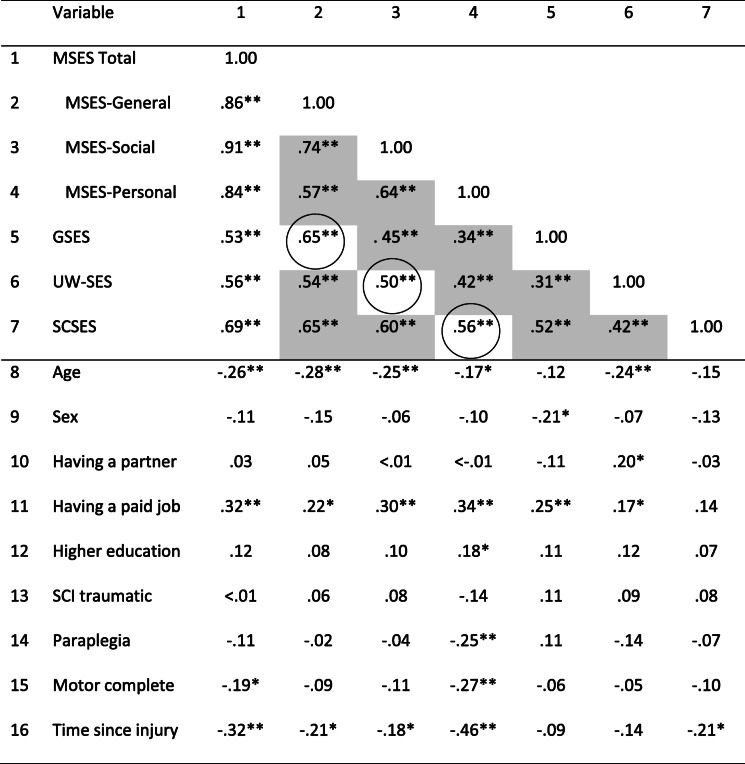
Pearson Product Moment correlation coefficients between the four self-efficacy scales or subscales, and participant characteristics

Abbreviations: *MSES total* Moorong Self-efficacy Scale total score, *MSES-General* Moorong Self-efficacy Scale General factor, *MSES-Social* Moorong Self-efficacy Scale Social functioning self-efficacy factor, *MSES-Personal* Moorong Self-efficacy Scale personal functioning self-efficacy factor, *GSES* General Self-efficacy Scale, *UW-SES* University of Washington Self-efficacy Scale, *SCSES* Self-care Self-efficacy Scale, *SCI* Spinal Cord Injury. * p < 0.05, ** *p* < 0.01 according to Pearson correlation analyses

All the SE scales showed moderate to strong significant inter-correlations. The first hypothesis that predicted strong inter-correlations would occur between scales that measure the same aspect of SE was confirmed, with correlations between 0.50 and 0.65 being found (circled in Table 3). However, the hypothesis that SE scales that measure general SE (MSES-General and GSES) versus domain-specific SE (MSES-Social and UW-SES) versus task-specific SE (MSES-Personal and SCSES) would show moderate, but not strong inter-correlations was not confirmed. Over 50% (7 out of 12) of the inter-correlations were strong (shaded in Table 3). Strong correlations were found for the MSES-General with all scales/subscales, for MSES-Social with MSES-Personal, MSES-Social with SCSES, and GSES with SCSES.

## Discussion

The hypotheses concerning the inter-correlations between the four SE scales could not be fully confirmed. All scales measuring the same aspects of SE (i.e., general, domain-specific, or task-specific SE) had strong inter-correlations. However, over half (7 out of 12) of the correlations between scales measuring different aspects of SE were strong as well. This result suggests the presence of a common latent construct underlying all four scales. From the current study, it is not clear whether the general SE scales also measure state aspects or the other way around. Inspection of the separate questions suggests that it is not very likely that the trait scales measure state aspects of SE. On the other hand, in the scales measuring state aspects, more general aspects could be distinguished; for instance, question six of the UW-SES-6: ‘You can figure out effective solutions to spinal cord injury-related issues that come up?’

The results therefore indicate that it is difficult and perhaps clinically confusing to measure state aspects of SE, and that the current “state” questionnaires incorporate trait aspects. It could also be argued that the distinction between trait and state SE is somewhat arbitrary and theoretical. In addition, the findings of the present study reflect the complex nature of the four SE scales, their factors and items that may incorporate various aspects of physical function, interpersonal relations, social participation, health and psychological wellbeing.

If both state and trait SE questionnaires tap into a core underlying trait construct it would, from a theoretical point of view, be more difficult to see changes over time in response to adjustment changes following SCI. A study using the GSES showed no overall changes in mean scores during inpatient rehabilitation, although there were some changes within individual participants [46]. Another study showed similar GSES scores at discharge and at 5 years post-discharge from initial inpatient rehabilitation [47]. Previous research has shown some variation over time in the MSES total score from admission to discharge from SCI rehabilitation, and 6 months post-discharge [16, 48]. Most participants in a recent study by Craig et al. [16] followed a stable trajectory and only a small percentage showed a decreasing trajectory, however, it is not clear if this change over time is of statistical significance. Another study with the MSES did not find a significant change between admission and discharge [49]. A study using the Self-Efficacy for Managing Chronic Disease Scale, like the MSES measuring trait and state aspects of SE, also did not find any change from inpatient rehabilitation and 3-months post discharge. Furthermore, a study that used the self-rated Abilities for Health Practice Scale, that also measures both trait and state aspects of SE, did not find any change in scores after an intervention [50]. Only in one study, about wheelchair skills training was a significant increase in task-specific wheelchair use SE found [51]. To date, very few SE scales used in SCI research appear sensitive enough to detect change over time, and the results of this study suggest strongly that this is because most SE scales tap into this latent underlying general construct or trait aspects of SE rather than domain-specific of task-specific (state) aspects of SE.

Some researchers have raised the issue of whether SE scales actually measure SE rather than motivation [52]. By asking participants to indicate whether one “can do” a specific target behavior, one may unintentionally also measure aspects of motivation instead of SE. Williams and Rhodes argued that controlling for motivation by adding the phrase “if you wanted to” to each question could decrease the SE scale tapping into motivational issues [52]. Such a change in SE scale instructions could improve the assessment of SE in future research, which may clarify our understanding of the specific features of state SE and how this concept can be assessed validly and be sensitive enough to measure change in a person’s life or after receiving an intervention.

Nonetheless, SE is a central and crucial concept in rehabilitation psychology, and is predictive of adjustment after SCI [9, 17, 53]. These studies were conducted using scales that appear to measure a combination of trait and state aspects of SE. One could argue that scales that explicitly measure state aspects of SE, in which domain-specific knowledge and skills are needed to meet the challenges associated with living with a SCI, would be even more predictive [15]. However, it is also possible that general SE may moderate the impact of certain environmental factors. For example, if someone believes that they can successfully execute a behavior required to produce a desired outcome, in day to day situation (this is general SE) that same person might also believe they are able to achieve such an outcome in more specific tasks within a specific context (that is domain-specific or task-specific SE). In terms of improved measurement, it has been suggested that custom-made short-forms that measure different levels of SE (e.g., high vs low) may provide greater precision, with the potential to develop separate norms [32]. Within the SCIAM model, a division is made between trait aspects of SE, as part of the psychological factors, and state aspects of SE, as part of the appraisal and reappraisal process, the so called “engine room”. The performance of this latter process is seen as crucially important for adjustment and therefore even more important for predicting adjustment following SCI. It is hoped this study will help improve the clinical assessment of SE in the appraisal-reappraisal process.

This study knows some limitations, first, in this study a relatively small sample size was used, and for this reason the interpretation of the results must be approached with some caution. Secondly we used cross sectional data, 6 months after initial rehabilitation. We do not know how the inter-correlation will develop over time. Further, we combined cohorts from two countries, while largely the same they had different sample sizes and different language and cultures that might have had some influence on results. Lastly, the SCSES was modified from the Managing Disease in General Subscale of the Self-efficacy for Managing Chronic Disease Scale and being a new scale had not been validated prior to this study. However, the results presented in this study have shown the adapted scale has construct validity.

## Conclusions

The expected distinction between SE as a trait versus a state construct involving different aspects (general, domain-specific and task-specific) could not be demonstrated. This result suggests the presence of a common latent construct underlying all four scales, most likely general aspects of SE. Further research is necessary to find ways to improve the measurement of domain-specific and task-specific aspects of SE, so that they are sensitive enough to capture change over time, and thus enhance clinical outcomes of people with SCI as they adjust to their disability.

## Supplementary information

**Additional file 1: Supplementary file 1.** Self-care Self-efficacy Scale. The introduction and five questions of the Self-care Self-efficacy Scale.

## Data Availability

The datasets used and/or analysed during the current study are available from the corresponding author on reasonable request.

## Abbreviations

GSES: General Self-efficacy Scale
MSES: Moorong Self-efficacy Scale
MSES-General: Moorong Self-efficacy Scale General self-efficacy subscale
MSES-Personal: Moorong Self-efficacy Scale Personal functioning self-efficacy subscale
MSES-Social: Moorong Self-efficacy Scale Social functioning self-efficacy subscale
SCSES: Self-care Self-efficacy Scale
SE: Self-efficacy
SCI: Spinal cord injury
UW-SES: University of Washington Self-efficacy Scale
