# Supplementary material for: Enhancing our conceptual understanding of state and trait self-efficacy by correlational analysis of four self-efficacy scales in people with spinal cord injury
Source: BMC Psychol. 2020 Oct 19;8:108. doi: 10.1186/s40359-020-00474-6 (PMC7574195; doi:10.1186/s40359-020-00474-6)
Supplement: Supplementary file 1 — Additional file 1: Supplementary file 1. Self-care Self-efficacy Scale. The introduction and five questions of the Self-care Self-efficacy Scale. [file 40359_2020_474_MOESM1_ESM.docx]

**Supplementary files**

**File Name:** Supplementary file 1

**Title:** Self-care Self-efficacy Scale

**Description:** The introduction and five questions of the Self-care Self-efficacy Scale

**Confidence in self-management**

**Having a spinal cord injury may mean that extra attention is needed for health to prevent physical problems. In the following questions please state how much confidence you have, where 0 = not at all confident at all and 10 = completely confident.**

| 1. | How confident are you that you can do all the things necessary to manage your condition on a regular basis? | | | | | | | | | | | | |
| --- | --- | --- | --- | --- | --- | --- | --- | --- | --- | --- | --- | --- | --- |
| Not at all  confident | |  | | | | | | | | | | | Completely confident |
|  |  | 0 | 1 | 2 | 3 | 4 | 5 | 6 | 7 | 8 | 9 | 10 |  |
|  |  |  | | | | | | | | | | |  |

| 2. | How confident are you that you can judge when the changes in your illness mean you should visit a doctor? | | | | | | | | | | | | |
| --- | --- | --- | --- | --- | --- | --- | --- | --- | --- | --- | --- | --- | --- |
| Not at all  confident | |  | | | | | | | | | | | Completely confident |
|  |  | 0 | 1 | 2 | 3 | 4 | 5 | 6 | 7 | 8 | 9 | 10 |  |
|  |  |  | | | | | | | | | | |  |

| 3. | How confident are you that you can keep up your physical condition and weight? | | | | | | | | | | | | |
| --- | --- | --- | --- | --- | --- | --- | --- | --- | --- | --- | --- | --- | --- |
| Not at all  confident | |  | | | | | | | | | | | Completely confident |
|  |  | 0 | 1 | 2 | 3 | 4 | 5 | 6 | 7 | 8 | 9 | 10 |  |
|  |  |  | | | | | | | | | | |  |

| 4. | How confident are you that you can prevent problems like pressure sores or urinary tract infections? | | | | | | | | | | | | |
| --- | --- | --- | --- | --- | --- | --- | --- | --- | --- | --- | --- | --- | --- |
| Not at all  confident | |  | | | | | | | | | | | Completely confident |
|  |  | 0 | 1 | 2 | 3 | 4 | 5 | 6 | 7 | 8 | 9 | 10 |  |
|  |  |  | | | | | | | | | | |  |

| 5. | How confident are you that you can do everything necessary, in order to get the right aids and medication? | | | | | | | | | | | | |
| --- | --- | --- | --- | --- | --- | --- | --- | --- | --- | --- | --- | --- | --- |
| Not at all  confident | |  | | | | | | | | | | | Completely confident |
|  |  | 0 | 1 | 2 | 3 | 4 | 5 | 6 | 7 | 8 | 9 | 10 |  |
|  |  |  | | | | | | | | | | |  |
